# Making sense of “STEM education” in K-12 contexts

**DOI:** 10.1186/s40594-018-0127-2

**Published:** 2018-08-24

**Authors:** Tamara D. Holmlund, Kristin Lesseig, David Slavit

**Affiliations:** Washington State University, 14204 NE Salmon Creek Avenue, Vancouver, WA 98686-9600 USA

## Abstract

**Background:**

Despite increasing attention to STEM education worldwide, there is considerable uncertainty as to what constitutes STEM education and what it means in terms of curriculum and student outcomes. The purpose of this study was to investigate the commonalities and variations in educators’ conceptualizations of STEM education. Sensemaking theory framed our analysis of ideas that were being selected and retained in relation to professional learning experiences in three contexts: two traditional middle schools, a STEM-focused school, and state-wide STEM professional development. Concept maps and interview transcripts from 34 educators holding different roles were analyzed: STEM and non-STEM teachers, administrators, and STEM professional development providers.

**Results:**

Three themes were included on over 70% of the 34 concept maps: interdisciplinary connections; the need for new, ambitious instructional practices in enacting a STEM approach; and the engagement of students in real-world problem solving. Conceptualizations of STEM education were related to educational contexts, which included the STEM education professional development activities in which educators engaged. We also identified differences across educators in different roles (e.g., non-STEM teacher, administrator). Two important attributes of STEM education addressed in the literature appeared infrequently across all contexts and role groups: students’ use of technology and the potential of STEM-focused education to provide access and opportunities for all students’ successful participation in STEM.

**Conclusions:**

Given the variety of institutionalized practices and school contexts within which STEM education is enacted, we are not convinced that a single worldwide definition of STEM education is critical. What we do see as essential is that those working in the same system explore the common elements that are being attributed to STEM education and co-construct a vision that provides opportunities for all their students to attain STEM-related goals. This is especially important in the current reform contexts related to STEM education. We also see that common conceptions of STEM education appear across roles and contexts, and these could provide starting points for these discussions. Explicitly identifying the ideas educators are and are not selecting and retaining can inform professional learning activities at local and larger scales.

Across the world, STEM receives tremendous attention in education reform efforts and in popular media. The International Council of Associations for Science Educators (ICASE 2013) recently urged member countries to work together to improve access to, and the quality of, STEM education in order to prepare all students for global citizenry. In the USA, the National Science Foundation (NSF) has played a significant role in the STEM education movement by calling for research related to science, mathematics, engineering, and technology. While the NSF first used the term “SMET,” this was revised into the more euphonic “STEM” in the early 2000s (Patton 2013). Shortly thereafter, the US government issued several studies on the state of STEM learning, and the number of schools designated as STEM-focused increased. Numerous legislative actions also emerged at this time related to computer science, STEM teachers, and STEM as career and technology (CTE) education (Gonzalez and Kuenzi 2012; Kuenzi 2008).

The NSF continues to use the STEM as an overarching title—for example, in requests for proposals—and activity within any one of the four disciplines can fit into the STEM category. For example, engaging elementary children in engineering and design, developing middle-level mathematics curriculum, or studying high school biology students’ understandings about evolution are all STEM activities. However, in the general public and among K-12 educators, “STEM education” is being increasingly viewed as a new concept, one that somehow brings all four disciplines together. One definition that illustrates an integrated perspective of STEM education comes from work in southwest Pennsylvania:

STEM education is an interdisciplinary approach to learning where rigorous academic concepts are coupled with real world lessons as students apply science, technology, engineering, and mathematics in contexts that make connections between school, community, work, and the global enterprise enabling the development of STEM literacy and with it the ability to compete in the new economy. (Southwest Regional STEM Network 2009, p. 3)

Despite the increasingly common use of the term “STEM education,” there is still uncertainty as to what constitutes STEM education and what it means in terms of curriculum and student outcomes (Breiner et al. 2012; Lamberg and Trzynadlowski 2015). STEM education can be considered a single or multi-disciplinary field, and in the case of the latter, no clear consensus exists on the nature of the content and pedagogic interplay among the STEM fields. While science and mathematics education are well-defined (though separate) entities across elementary and secondary schools worldwide, engineering education has largely been a function of higher education in the USA. And technology education has traditionally been delegated to vocational education (now called CTE), when included at all in secondary schooling. Given that policymakers, parents, and business communities are calling for STEM education across grade levels and that STEM literacy is viewed as critical for the economic success and health of individuals and nations worldwide (National Science Board 2015; STEM Education Coalition 2014), it is important to consider the varied meanings that different groups may have for STEM and STEM education. While it may not be necessary, or even feasible, to coalesce around one common definition of STEM education, we argue that without some shared understandings across a system, it is difficult to design and implement curriculum and instruction to promote successful STEM learning for all students.

In this study, we investigated the conceptualizations of STEM education among educators who work in STEM-focused settings. Our analysis centered on identifying the themes that arise in these educators’ conceptualizations. We also looked for possible relationships between these conceptualizations and (a) their professional work context, including relevant supports for professional learning (referred to as *context group*), as well as (b) their professional roles (referred to as *role group*).

## Conceptualizing STEM education

Consistent with many international recommendations, two National Research Council (NRC) reports on successful K-12 STEM programs in the USA described three major and inclusive goals for STEM education: (a) increase the number of STEM innovators and professionals, (b) strengthen the STEM-related workforce, and (c) improve STEM literacy in all citizens (National Research Council 2011a, 2013). But what does it mean, at the classroom level, to implement STEM education? Current research suggests that STEM education is an *innovation* with various instructional models and emphases that are shaping reform in many educational systems (Bybee 2013; National Academy of Engineering and National Research Council 2014; Wang et al. 2011). Emerging research shows a lack of consensus on the content and instructional practices associated with STEM education, with various models being promoted. These include the incorporation of an engineering design process into the curriculum (Lesseig et al. 2017; Ring et al. 2017; Roehrig et al. 2012), a thematic approach centered around contemporary issues or problems that integrates two or more STEM areas (Bybee 2010; Zollman 2012), and maker-oriented programs such as robotics, coding, and Maker Faires, which may occur outside of the regular school curriculum (Bevan et al. 2014)).

However, while various models have emerged, an analysis of STEM education does reveal an emerging consensus on the global attributes associated with this innovation. For example, Peters-Burton et al. (2014) compiled ten “critical components” of STEM high schools, and LaForce et al. (2014) identified eight “core elements” of STEM schools. At the classroom level, Kelley and Knowles (2016) provide a conceptual framework for secondary STEM education efforts. As these and other reports informed the content of the professional development for the participants in this study and our a priori coding categories, we next provide brief descriptions of common elements of STEM education.

One significant attribute of STEM-focused schools is the attention to instructional practices that actively engage and support all students in learning rigorous science and mathematics (Kloser 2014; LaForce et al. 2014; Lampert and Graziani 2009; Newmann and Associates 1996). These instructional practices are beginning to be known as a core or ambitious teaching (Kloser 2014; Whitcomb et al. 2009), and professional development that helps teachers develop these practices along with disciplinary content knowledge is often recommended for STEM-focused learning contexts. Other attributes of STEM-focused schools are student learning experiences that incorporate multiple disciplines (an interdisciplinary, integrated, or trans-disciplinary approach) and often include a project- or problem-based approach tied to authentic or real-world contexts (LaForce et al. 2014; Peters-Burton et al. 2014). Inherent in problem- and project-based learning are opportunities for student growth in twenty-first century skills such as collaboration, critical thinking, creativity, accountability, persistence, and leadership (Buck Institute 2018; Partnership for 21st Century Skills 2013). These projects often encompass partnerships with STEM professionals and other community members who can help students make connections between school learning, problem solving, and careers. Another important attribute is students’ use of appropriate and innovative technologies in their inquiries, research, and communication. In this study, we explore the extent to which these characteristics or any others were part of educators’ conceptions of STEM education.

## Research questions

Our interest in how educators conceptualize STEM education is grounded in our research on STEM schools and our participation as STEM professional development providers. We framed our study around the following question:

What sense have educators made of STEM education after implementing and/or supporting STEM learning experiences?

We were also interested in possible relationships between participants’ professional work contexts or professional roles and the themes they associated with STEM education. Thus, we addressed the following sub-questions in our analysis:

A. What themes emerge in the conceptualizations of STEM education among educators in a given professional context? What relationships might exist between an individual’s conceptualization of STEM education and the professional context in which she/he works?
B. What themes emerge in the conceptualizations of STEM education among educators in a given role group? What relationships might exist between an individual’s conceptualization of STEM education and his/her professional role?

## Theoretical framework

Understanding the intentions of reform proposals requires implementers to interpret what is meant and foresee implications on curriculum and instruction (Spillane et al. 2002). Because we are interested in the ways in which individuals are navigating the complex and novel ideas inherent in STEM education, we use sensemaking as our theoretical framework. Sensemaking theory attends to both the individual processing and the socially interactive work that occurs when a person encounters a gap in or discontinuity between what exists and a proposed change or innovation (Dervin 1992). Grounded in cognitive learning theory, sensemaking is a dynamic process where each person draws upon existing knowledge, beliefs, values, experiences, and identity to accommodate or assimilate new concepts (Weick 1995).

**Fig. 1:**
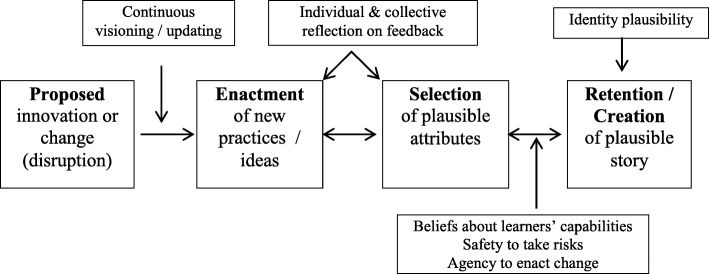
Sensemaking cycle (adapted from Weick et al. 2005, p. 414)

Sensemaking begins with a real or perceived disruption to the status quo, which may range from a fairly routine change, such as a schedule revision, to radical innovation in curriculum and instruction. Sensemaking involves a continuous cycle of enacting actions to address the disruption, noticing and categorizing aspects of the enactment, selecting elements that are plausible, and retaining those in future actions (see Fig. 1). Feedback from multiple sources shapes all these processes (Weick et al. 2005). The creation of a “plausible story” (Weick et al. 2005, p. 410) provides the implementer a way to reconcile the varied requirements, standards, and other ideas associated with a proposal for change within their current situation.

Sensemaking is situated within social and contextual components that influence the individual (Coburn 2001; Spillane et al. 2002). Any one person’s conceptualization can be “talked into existence” (Weick et al. 2005, p. 413), as it is shaped through dialog with others, by the constraints and affordances of the environment, and sometimes by the influence of leaders. Both individual and collective sensemaking can result in a range of meanings. While multiple perspectives are useful in generating ideas, this can also be problematic in terms of how new ideas are implemented. For example, there are numerous accounts of the challenges inherent in translating educational innovations or policies for reform into mathematics and science classrooms due to contrasting vision (Allen and Penuel 2015; Fishman and Krajcik 2003; Spillane 2001). Therefore, in this study, we are most interested in the *current status and result* of the participants’ sensemaking process rather than documenting the sensemaking process itself. Understanding how various stakeholders conceptualize new curricular or instructional ideas can inform the conversation needed to support professional learning and alleviate challenges to reform.

## Research design

“The assumptions and propositions of sensemaking, taken together, provide methodological guidance for framing research questions, for collecting data, and for charting analyses” (Dervin 1992, p.62). To understand what sense participants made of the information encountered and experiences they had about STEM education, we elicited each participant’s thinking through concept maps and interviews. Concept maps can show the “structure of knowledge” (Novak 1995, p. 79) by making explicit one’s ideas within a specific domain. Map creators identify ideas associated with the given domain and arrange these in a way to designate which are most salient and which are related but less significant. These main and subordinate ideas are called “nodes.” Connecting lines, arrows, and words written on these connecting lines can be used to show the interrelationships between the major and less significant nodes (Novak and Cañas 2008). As learning is contextual and informed by a learner’s previous knowledge (Bruner 1990), any two concept maps typically differ in multiple ways.

Concept maps have been used in K-20 education and in professional development to provide insight into how learners are structuring new ideas with existing understandings (Adesope and Nesbit 2009; Besterfield-Sacre et al. 2004; Greene et al. 2013; Markham et al. 1994). The act of map creation requires reflection on events, experiences, and ideas and, thus, is a sensemaking activity: “How can I know what I think until I see what I say?” (Weick et al. 2005, p. 416). In addition, concept maps allow participants time to make sense of what they think. The maps can then be used in interviews to provide focal points for further sensemaking (Linderman et al. 2011), with opportunities for the creator to elaborate and clarify the components and structures of the map.

While sensemaking is ultimately individualistic, the ideas and experiences that contribute to this occur in the context of organizations, conversation, shared activity, and feedback loops (Weick et al. 2005).

Sense-making does assume that the individual is situated in cultural/historical moments in time-space and that culture, history, and institutions define much of the world within which the individual lives . . . the individual’s relationship to these moments and the structures that define them is always a matter of self-construction. (Dervin 1992, p. 67)

In line with this theoretical perspective, the unit of analysis for our study is the individual. We report on this analysis to answer our first research question. We also recognize that each individual has a professional role and is situated within particular institutional structures and cultures and that both the responsibilities of one’s role and the context inform one’s conceptualization of STEM education. As such, we also noted the roles of each participant and developed rich descriptions of the professional contexts in which participants worked. These descriptions, in conjunction with participants’ concept maps and interviews, allowed us to look for relationships among participants’ conceptualizations of STEM education, their professional contexts (sub-question A), and their professional roles and responsibilities (sub-question B).

### Participants

Thirty-four people participated in this study. Each was affiliated with STEM education endeavors in one of three *context groups*. Thirteen participants were teachers and administrators at an inclusive, STEM-focused secondary school (Ridgeview STEM Academy^1^). Another 12 were teachers from two traditional middle schools who participated in a 2-year professional development project that supported their implementation of engineering design challenges with their students. Nine were STEM educators and stakeholders participating as faculty in a statewide professional development (PD) institute designed to assist district or school teams with the creation of a STEM education implementation plan. The professional roles of each participant are shown in Table 1.

**Table 1:** Pseudonyms of participants by context group and by professional role

Context group ➔ professional role	Ridgeview STEM Academy	Traditional Middle Schools	Statewide professional development faculty
Math teacher	Joan, Greg	Nina, Heather, Olivia, Denise, Regan	Carlton
Science teacher	Hunter, Clint	Anthony, Helen	–
Technology, Engineering, CTE teacher	Rachel, Brittany, Jad, Josh	Shawn, Beth	–
Non-STEM teacher	Monica, Jason	Petr, Pamala, Brenda	–
School/district administrator	Michelle, Will, Sandra	–	Marion, Bridget
Business/organization partner	–	–	Sophie, Abel, Hugh
Regional STEM educator	–	–	Janis, Claudia, Richard

Some participants in each context group held dual roles (e.g., Shawn was both a science and an engineering/CTE teacher; Will and Michelle were both non-STEM teachers and administrators). For the purpose of this analysis, the role they most strongly identified with at the time they completed the concept map was used to determine the *role groups*. The selection of these participants from the larger pool of teachers and administrators at all three schools was based on their participation in two larger research projects. The professional development faculty were included as participants to provide data from a group with very different contexts and, possibly, perspectives. Given the frequent lack of communication and difference in vision among groups associated with reform efforts (Spillane et al. 2002), it was important to get a snapshot of the thinking of a group situated outside of classrooms and schools.

### Professional work contexts

We describe the professional work contexts of each of our participants. With regard to the participants from Ridgeview STEM Academy and from the two traditional middles schools, we focus on the characteristics of the school and the supports teachers received for their professional learning. In the case of the statewide PD faculty, we focus primarily on their leadership roles in the context of a statewide STEM education leadership institute.

#### Ridgeview STEM Academy

The participants in this context group were from Ridgeview STEM Academy (RSA), an inclusive STEM-focused school that opened in 2012 with nine teachers and students in grades 6, 7, and 9. The student population was intended to mirror the demographics of the district, and admission was obtained through a lottery by zip code. During the focus year of this study, RSA had approximately 400 students in grades 6–12 and 22 teachers. District-provided professional development associated with learning about the school vision, culture, and practices has been provided since the opening of the school, but teachers have predominantly made sense of STEM education as they implement it. The RSA vision statement described the student learning experience as one that would support the student as a “learner, collaborator, designer, and connector” and the faculty nurtured the growth of a school identity as a place where students had “voice and choice.” STEM learning was viewed as possible for all students, and the curriculum was envisioned as a project- or problem-based (Buck Institute 2018) and connected to “the real world of business and research.”

Teachers collaborated across the school year to develop their own interdisciplinary, project-based curricula and used overarching themes to integrate the humanities and STEM disciplines. They accessed a variety of resources as they experimented with the types of instructional practices needed to enact the school vision in the context of the Common Core State Standards (CCSS) (National Governors Association 2010) and the Next Generation Science Standards (NGSS) (Achieve 2013). Teachers explicitly supported building student skills and attitudes, such as persistence in problem solving, curiosity and a willingness to learn from failure, creative thinking, and the ability to work independently and collaboratively. The technology received attention from the start. Each student was provided a laptop loaded with design, research, and communication tools, and the school offered specific classes dedicated to the use of this technology. Bringing STEM professionals into the school and taking students out to explore STEM careers and work was an explicit focus, with a half-time position created to develop partnerships to support this. The administration assisted teachers in curriculum development by encouraging curricular risk-taking and continuous improvement.

The first and third authors conducted research at this school over a 5-year period (Slavit et al. 2016). We invited teachers who participated in our long-term study on STEM schools to participate in this investigation about sensemaking of STEM education. Interviews for this study were conducted with 13 RSA teachers and administrators over an 18-month period.

#### Traditional Middle Schools (TrMS)

This context group was composed of teachers from two middle schools (Rainier and Hood) in a large suburban school district. Both schools had traditional approaches to education, including a seven-period day and distinct courses for each content area (e.g., physical science, algebra, state history). Limited structures for teacher collaboration existed, and teachers’ interactions typically were by discipline and grade level. Each school had approximately 850 students in grades 6–8; 50% of these students came from low-income households, as determined by their qualification for free or reduced-price lunch.

Thirty-four science, mathematics, special education, and English language teachers from these two schools participated in *Teachers Exploring STEM Integration* (TESI), a 2-year professional development project that included a 2-week summer institute and ongoing support throughout the school years. Twelve of the 34 teachers participated in this study. TESI focused on the integration of STEM design challenges (DCs) into the existing middle school curriculum (Lesseig et al. 2016). An interdisciplinary team composed of scientists, mathematicians, and educators from a local university, community college, and school district developed several DCs that could be incorporated into the district’s existing mathematics and science curricula. The professional learning experiences in TESI were explicitly designed to model integrated STEM curricula aligned with math, science, and ELA standards. Authentic mathematical, scientific, and engineering practices received specific and ongoing attention, especially the identification and clarification of the problem; the importance of research, solution testing, failure, and feedback; and the development of evidence-based explanations. Teachers were provided with the literature about and video examples of core instructional practices (e.g., https://ambitiousscienceteaching.org) specific to mathematics and science. Teachers were also supported in making sense of an engineering design cycle and reflecting on the attributes of a strong design challenge in relation to the student learning experience. The need for and value of STEM learning was also contextualized in terms of twenty-first century challenges and opportunities for innovation.

During the first week of each summer session, teachers engaged in STEM DCs to support their learning about the relevant disciplinary content and to gain familiarity with the engineering design process. During the second summer week, middle school students identified by their teachers as struggling in mathematics or science were invited to attend each morning session; teachers worked alongside the students to solve a design challenge. Engineering, mathematics, and science professors from the university and a variety of other professionals (e.g., a prosthetics designer, a government climate scientist) interacted with the teachers and students. Teachers spent the afternoons reflecting on the students’ engagement, analyzing instructional practices, and planning for the implementation of design challenges in their classrooms. While undertaking design challenges, teachers and students were involved in collaborative and creative problem solving, communication, and critical thinking. The use of various forms of technology was modeled during the professional development summer institutes. The recognition that every student could be a successful contributor to solving a design challenge was also an explicit element of the TESI project.

The second author was the PI for TESI, and the other two authors were involved in the planning and advisory committees. The teachers interviewed for this study were in the TESI project for 2 years and also participated in a study of the implementation of ideas from that project. Participants from one school included eight eighth-grade mathematics, science, STEM, English as a second language, and special education teachers. Participants from the second school included four sixth-grade teachers of mathematics, science, STEM, and special education (see Table 1). All were interviewed in the fall of the second year of the project.

#### Statewide professional development faculty

The professional work context for each of these nine participants was different than that of the TrMS and RSA educators. All shared a common experience as leaders in a statewide STEM education leadership institute. Yet, each came from a different professional context, and they collectively held a variety of professional roles (see Table 1).

The PD faculty were responsible for developing and implementing a week-long summer institute on STEM education and leadership for school and district teams from across the state. The institute focused on the development of and leadership for an implementation plan for STEM education. The content of the institute was grounded in the NGSS, CCSS, and CTE standards (https://careertech.org) and informed by the NRC (2011a, 2011b) reports on STEM education. In addition, each faculty member brought a wealth of expertise relevant to STEM education from their professional roles external to this initiative; for example, one was a principal of an elementary STEM school and another a scientist at a national laboratory (see Table 1). Many were involved with science and mathematics PD at local and regional levels. Across the year, these institute faculty members developed a list of relevant resources that could be useful to institute participants, including model STEM schools, websites, research and practitioner literature, curricula, and STEM activities. Across multiple meetings, the faculty drew upon these resources and their own expertise to develop sessions for the summer institute. Various sessions focused on the meaning and value of STEM education, including how to integrate isolated school subjects and provide connections to the real-world needs and careers, the importance of partnerships between STEM educators and STEM professionals, equity in STEM learning opportunities, and how to anticipate and address common challenges associated with change. Thus, preparation for and implementation of the various sessions in this institute provided opportunities for all faculty members to share their expertise and clarify key ideas about STEM education.

The PD faculty were invited to participate in this study as their perspectives give us insight into how educators who are promoting the innovation are conceptualizing it, what they identify as important, and the extent to which the messages they convey are coherent and consistent. They created their concept maps during the first of a 2-day planning meeting for the summer STEM education institute. The first author was a member of this faculty and had worked with all but two of the members for at least 5 years.

### Data collection

Based on our long-term work within each of the three contexts, we had in-depth information about the STEM-relevant contexts for each of the three participant groups, and the actions group members were asked to take. The above descriptions of each of these contexts were developed in order to address sub-question A about potential relationships between contextual elements and participants’ sensemaking about STEM education.

**Fig. 2:**
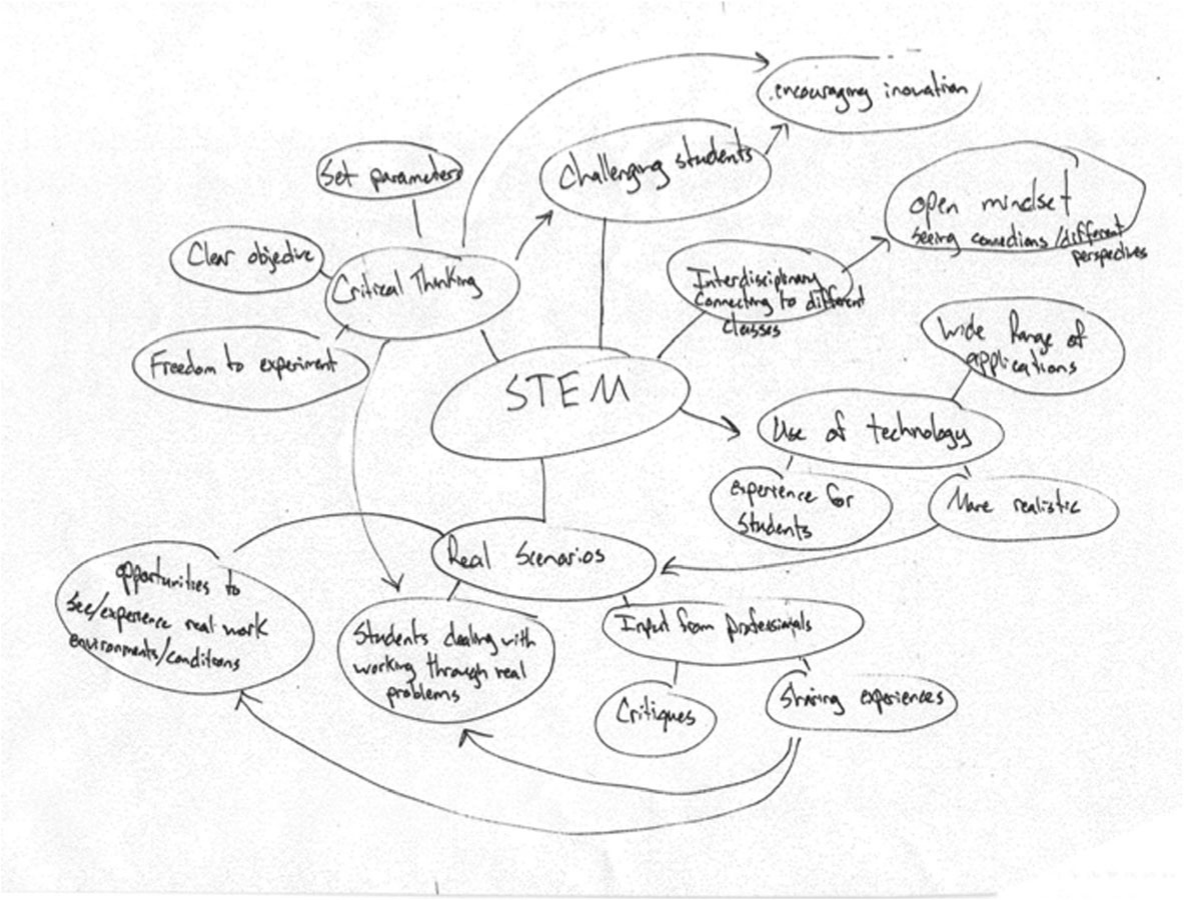
Hierarchically arranged concept map from Hunter, RSA

**Fig. 3:**
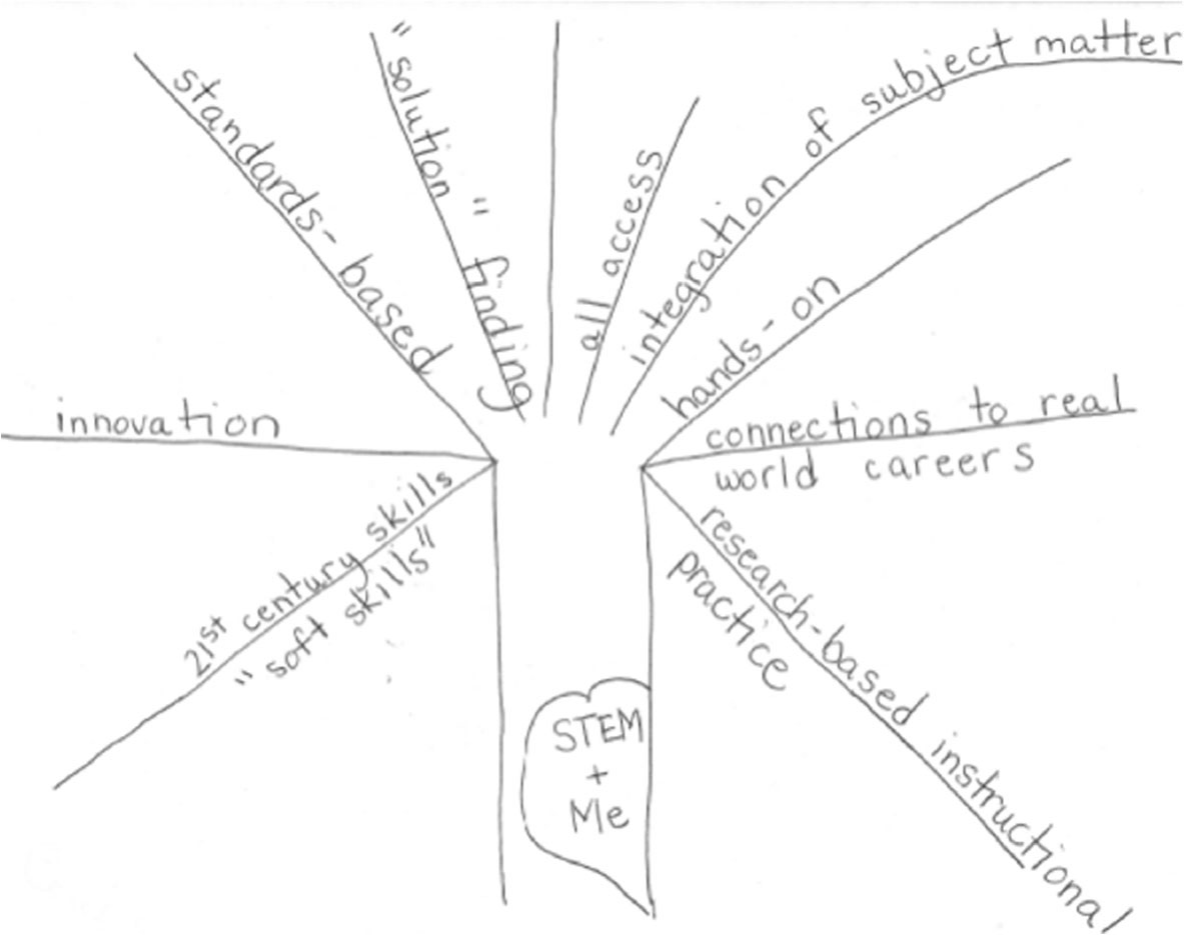
Non-traditional concept map, Bridget, PD faculty

To capture participants’ conceptualizations of STEM education, we asked them to construct concept maps and used follow-up interviews to clarify the meaning of map elements. At the time of the interviews, each participant had implemented some kind of STEM education-related action multiple times and had opportunities to individually and collectively make sense (envision, enact, select, retain) of STEM education. Initially, each participant was asked if they were familiar with concept mapping and, if needed, given a brief overview about representing concepts and sub-concepts hierarchically. They were asked to construct a concept map in response to two questions: “What is your understanding or conception of STEM education? and What do you see as the most important ideas and sub-ideas?” Due to contextual constraints, participants created their concept maps in varied settings. The participants from the three schools were invited to meet with researchers in pairs or individually at a time convenient to them. The PD faculty developed their concept maps individually while all were in the same room. Each person was given as much time as needed to develop her/his map. The researcher read or wrote while participants were constructing their maps to alleviate potential discomfort. Participants were not held to using a traditional hierarchical structure in their mapping; as such, map formats ranged widely (Figs. 2 and 3).

After concept mapping, semi-structured interviews were used to provide participants with another opportunity to make sense of their ideas about STEM education and inform the research findings. TrMS and RSA participants were interviewed immediately after constructing their maps. Due to time constraints, clarification of the maps of the PD faculty was done informally over the duration of the faculty meeting rather than with semi-structured interviews. However, for three PD faculties who constructed non-traditional concept maps (e.g., Fig. 3), semi-structured telephone interviews were conducted. Participants were asked to “talk us through” their concept maps. The interviewer would then follow up on a particular idea or ask a participant to elaborate on specific ideas they brought up. The researcher also asked what, if any, questions participants had about STEM education, and what supported them in coming to these particular views of STEM education. In cases where interviews were conducted in pairs, participants were asked to compare and contrast their maps or to comment on specific ideas that may have appeared on a colleagues’ map. For some participants, the interview prompted them to make modifications to the map or express additional ideas that were not on the map. For others, the interview did not result in additional information. In explaining the components of the map, participants could notice what they had included (or not) and how they had portrayed relationships between ideas.

### Data analysis

#### Overview

Concept maps can be analyzed quantitatively and qualitatively (Greene et al. 2013). A quantitative analysis involves counting nodes (concepts), hierarchies (chains of sub-concepts out of one node), and cross-links between hierarchies to infer the complexity of the map creator’s understanding of the concept being represented. However, because we allowed each participant to represent their thinking in whatever way it made personal sense, some of the participants’ maps did not readily translate to quantitative analyses (e.g., did not include identifiable nodes or were global in nature, see Fig. 3). We chose to analyze the concept maps qualitatively and analyzed the interview data concurrently to aid our interpretation of the concept maps. We looked at the maps holistically, attending to the overall structure, the words used as nodes, and words used as cross-links. These analyses led to our primary results on the participants’ views of STEM education, including the emergence of our themes, and a secondary quantitative synthesis of each theme’s frequency across the participants’ context groups and role groups.

#### Generating themes

We drew on current research on STEM education as well as a grounded approach based on our interviews with teachers to generate our initial themes (Breiner et al. 2012; LaForce et al. 2014; Peters-Burton et al. 2014; Sanders 2009). We developed nine initial themes and added three others as the coding progressed. The initial themes were a synthesis of the way participants represented or talked about the attributes of STEM education and the major attributes that are described across the literature. For example, because project- or problem-based learning (PBL) tends to be situated in real-world contexts, we originally had one theme for PBL that included real-world connections. However, on a majority of concept maps, there were distinct nodes for real-world problem solving and others for attributes that characterized the student learning experience, regardless of whether it was within a PBL approach. Thus, we created different themes for these two distinct aspects of STEM education (RWPS, StLE; see Table 2).

**Table 2:** Coding themes and rules

Code	Theme	Rules
IntDis	Interdisciplinary, cross-disciplinary, integrated curriculum	STEM-focused curriculum across two or more subjects or integration of technology and engineering into math and science core concepts; formal and informal extensions and connections to include writing, reading, social studies, etc.
InstPrac	Instructional practices necessary for developing and implementing STEM education learning experiences	About teachers’ planning, decision-making, in-the-moment actions, and reflections upon teaching and learning; what teachers do to engage students in learning: active participation, classroom discourse, voice and choice, student-centered instruction. Teachers’ awareness of the demand for more reform-informed or ambitious instructional practices.
Tech	Increased use of technology in the context of PBL or EDC	Including and beyond information and communication technology use. Do not code if the technology is just listed as part of STEM.
Stan	Standards and the disciplinary content and practices of math, science, engineering, other	References to NGSS, CCSSM, and CTE standards. May include concepts, practices, core content, set curriculum, scope, and sequence.
21CS	Twenty-first century skills	Opportunities for students to develop and practice skills and dispositions such as problem solving, collaboration, critical thinking, communication of ideas and results, creativity and innovation, and perseverance.
Prtnr	Professional partnerships	Connecting students with STEM professionals, into and outside of the classroom; building connections to careers and internships. Not about teacher collaboration.
Equ	Equity in opportunities to be successful in STEM learning	Access and opportunities to all the above for all students; thinking about who the child is, taking each student’s needs and strengths into account; recognizing the individuality of each student; seeing the particularities of a STEM education approach (teaching, learning, curriculum) as providing access for each student to participate, contribute, and grow.
RWPS	Real-world problem solving with integrated curricular themes	Authentic learning experiences and curricular connections between in-school tasks and out-of-school contexts. Curricular themes contextualized in real-world problems, issues, and needs.
StLE	Students’ learning experiences especially related to project- or problem-based learning (PBL) or engineering design challenges (EDC)	Attributes of students’ learning experiences; content or problem is meaningful to kids; sustained inquiry; authentic application of disciplinary practices and knowledge; rigorous content; use of technology; student-generated artifacts; presentations to the authentic audience; engineering design cycle, especially including empathy, research, failure, and redesign.
Val	Value of STEM	Developing STEM literate citizens, STEM literacy, global citizenship, and economic power.
TchNd	Teacher needs	Deep content knowledge in at least one of the STEM fields, pedagogical content knowledge, time for collaborative research and planning, and curricular knowledge.
ChPrb	Challenges and problems with STEM education	Lack of common understanding; lack of instructional resources; privileging math and science over humanities; politicization; attributes (above) out of alignment with school structures and instructional practices.

The three codes (Val, TchNd, ChPrb in Table 2) were added later in the coding process to better capture significant themes that emerged in our analysis. For example, when we began coding the concept maps of the PD faculty, we saw ideas that related to opportunities to practice twenty-first century skills through PBL but also referred more generally to creating a STEM-literate citizenry. Thus, we created a separate theme to capture this more global perspective. Specifically, we coded nodes that focused predominantly on the abilities and dispositions of each student to communicate, work collaboratively, think creatively, or persevere in problem solving as “twenty-first century skills” (21CS). Nodes that reflected a broader conceptualization related to global citizenship and STEM literacy as having economic and other societal benefits were recoded as “value of STEM literacy” (Val).

We also developed two themes to reflect nodes associated with the conditions needed for implementing STEM-oriented teaching or curriculum. Ideas associated with what teachers might need in order to implement STEM education such as content knowledge and time for collaborative planning were coded as “teacher needs” (TchNd). Challenges and problems in implementing STEM education (ChPrb) showed up on some concept maps or, more frequently, emerged during the interviews. These responses ranged from structural constraints, such as lack of collaborative planning time or students in one class not having the same mathematics and science teachers (preventing extending projects across two class periods), to the politicization of STEM education.

#### Thematic analysis

In January 2015, the first author analyzed each map from the TrMS and RSA participants, generating themes based on the words participants used as nodes (concepts and sub-concepts) and cross-links (e.g., a line labeled “supplement each other” drawn between the nodes for “science” and “math” would be coded as IntDis for integration). Coding rules were developed and used to clarify the coding themes. In August 2015, PD institute faculty maps were obtained and coded by the first author, and coding rules were further elaborated. In September 2015, the second two authors and a research assistant coded six concept maps. After discussions with the first author, coding rules were further clarified and made more specific, especially to distinguish between student learning experiences and instructional practices. To check the reliability of our thematic coding on complex, non-traditional maps, the first author conducted follow-up interviews with three of the PD faculty and found that the initial coding accurately represented the mapmaker’s intentions. Based on the revised and/or clarified coding rules, the first author recoded all 34 concept maps, using the interview transcripts and concept maps concurrently. As the interview protocol probed for explanations about each map element, the transcripts helped clarify meanings or validate interpretations of cross-links and nodes.

#### Quantifying the themes

We coded 34 concept maps as described above and then counted how many people included each theme in their concept maps. After all maps were coded for the themes, we counted the occurrence of each theme, recorded these for each individual, and compiled the total inclusion of each theme. This allowed us to respond to our main research question. To address our two sub-questions, we then looked at the frequency of theme inclusion for each context group (RSA, TrMS, PD faculty) and also determined the frequency of inclusion of each theme by general role groups: STEM teachers (18 secondary math, science, technology, engineering, CTE teachers), non-STEM teachers (5 secondary special education or ELL teachers), school or district administrators (5), and non-school-based external partners (6 partners from businesses or organizations or regional PD providers). We present a discussion of our analyses in the next section.

## Limitations

The use of concept maps to elicit conceptualizations of STEM education has multiple limitations. Although we allowed participants to construct their maps in non-traditional ways, including writing a paragraph instead of mapping, some may have felt uncomfortable portraying their ideas using this type of representation or may not have included all their ideas. While the interviews provided an opportunity for participants to add to or expand upon their representations, participants may have held ideas they did not want to share, lacked the ability or language to represent, or perhaps were not considering at the time of the interview. Moreover, participants may not have mentioned certain ideas they perceived as obvious, such as the inclusion of *all* students in STEM experiences. There are also limitations related to the participant pool. There were limited numbers of non-STEM teachers (5), administrators (5), and external partners (6) in comparison with the number of STEM teachers (18) who participated. However, the concept maps and interviews with all participants provide insight into the variation that is possible in making sense of STEM education.

## Results

We address our main research question by showing the frequency of the various themes relevant to STEM education (coding categories) that were included in individual concept maps (Table 3) and providing examples that show different individual’s conceptualizations of the theme at the time. We then address sub-question A by showing the frequency of theme inclusion by context group (Table 4) and examining relationships between the conceptualizations of STEM education and the context in which participants implemented STEM education activities. Finally, we address sub-question B by organizing the themes by role group (Table 5) and discussing potential relationships between the responsibilities inherent in specific roles and the elements of STEM education that surfaced in the concept maps among participants in that role. Our data suggest that certain aspects of STEM education are more salient in participants’ conceptions, and both context and role group contribute to these conceptions.

**Table 3:** Total number and percentage of participants who included each theme on concept maps or interview

Theme	IntDis	InstPrac	RWPS	StLE	21CS	Stan	Prtnr	ChPrb	Equ	Tech	Val	TchNd
Total (of 34)	29	25	24	20	18	14	13	12	10	10	6	6
% of total	85	74	71	59	53	41	38	35	29	29	18	18

*21CS* twenty-first century skills, *ChPrb* challenges and problems, *Equ* equity, *InstPrac* instructional practices, *IntDis* interdisciplinary, *Prtnr* partnerships, *RWPS* real-world problem solving, *Stan* standards, *StLE* student learning experience, *TchNd* teacher needs, *Tech* increased technology, *Val* value; (see Table 2 for more description)

**Table 4:** Frequency of inclusion of STEM education theme by participants in each context group

	Traditional Middle Schools (TrMS)	Ridgeview STEM Academy (RSA)	Statewide PD faculty
67–100%	IntDis, 100% InstPrac, 83% StLE, 67%	IntDis, 92% InstPrac, 77% RWPS, 77%	RWPS, 84% Prtnr, 67%
50–66%	Stan, 58% 21CS, 58% ChPrb, 58% RWPS, 50%	StLE, 62% 21CS, 54% Prtnr, 54%	IntDis, 56% InstPrac, 56% Val, 56%
33–49%	Equ, 33%	Tech, 38%	Tech, 44% 21CS, 44% Equ, 44% StLE, 44% Stan, 33%
0–32%	TchNd, 17% Tech, 8% Val, 0 Prtnr, 0	Stan, 31% TchNd, 23% ChPrb, 23% Equ, 15% Val, 8%	ChPrb, 22% TchNd, 11%

*21CS* twenty-first century skills, *ChPrb* challenges and problems, *Equ* equity, *InstPrac* instructional practices, *IntDis* interdisciplinary, *Prtnr* partnerships, *RWPS* real-world problem solving, *Stan* standards, *StLE* student learning experience, *TchNd* teacher needs, *Tech* increased technology, *Val* value; (see Table 2 for more description)

**Table 5:** Inclusion of themes by participants in each role group

Concept map themes with % inclusion overall, as shown in Table 3	STEM teachers (18), %	Non-STEM teachers (5), %	School or district administrators (5), %	External partners (6), %
Interdisciplinary (IntDis), 85%	89	100	80	67
Instructional practices (InstPrac), 74%	83	60	100	33
Real-world problem solving (RWPS), 71%	83	60	60	83
Student learning experiences (StLE), 59%	72	20	80	33
Twenty-first century skills (21CS) 53%	61	20	60	50
Standards (Stan), 41%	44	20	60	33
Partnerships (Part), 38%	28	20	60	67
Challenges and problems (ChPb), 35%	33	40	40	33
Equity (Equ), 29%	28	20	60	17
Technology (Tech), 29%	22	20	60	33
Value (Val), 18%	6	0	0	83
Teacher needs (TchNd), 18%	17	20	40	17

### Making sense of STEM education

We first tabulated the inclusion of theme by individuals and calculated the percentage of participants who included each theme. As shown in Table 3, there were three common themes on the concept maps: a connection across disciplinary subjects (IntDis), a focus on what teachers must attend to instructionally (InstPrac) when implementing a STEM approach, and explicit connections between in-school content and out-of-school problems or contexts (RWPS).

Interview data provided detail on how each participant conceptualized these themes. For example, when asked what her inclusion of the word “integration” meant (IntDis), a special education teacher from the TrMS group explained:

The reading, the writing, the art, the creativity. You know? You’re using computer skills. You’re using building skills. … So it makes [students] use everything. And the cool thing is they don’t know they’re using all that. (Brenda, interview, January 29, 2015)

A member of the PD faculty who was also the principal of an elementary STEM school talked about how real-world problems helped the teachers develop integrated curricula:

What we do is intentionally interweave the S, the T, the E, the M into instruction. So, at a typical elementary or middle school, often subjects are segmented and segregated, kind of siloed. Our commitment is that our students are doing STEM every day … . We intentionally plan STEM … we take the standards and cut them all apart and then piece them all together so we have consistent themes or overarching problems for students to solve. (Bridget interview, September 30, 2015)

A middle school science teacher from RSA also included real-world connections and instructional decision-making on his map. In his interview, he explained why real-world connections were important and how he developed these:

And so I started with real world scenarios, just because to me the science, technology, engineering and mathematics, kind of the end goal is getting students more fully prepared for real life. And so having them deal with real world scenarios helps them to do that. Couple of different ways to do that, one I had input from professionals … .And then opportunities to see and experience that real world, or real work, environment or conditions. (Hunter interview, November 4, 2014)

Participants represented these three themes (integration, real-world connections, and instructional practices) separately on their maps but, as seen by these comments, often revealed significant relationships among these themes in their interviews.

Over half of all participants included attributes of students’ learning experiences (StLE) and students’ opportunities to develop twenty-first century skills (21CS) as salient features of STEM education. Ideas related to the attributes of the student learning experience were represented on 59% of concept maps. Comments about this often addressed students’ engagement in the authentic practices of each discipline. A high school math teacher at RSA explained that “Kids should be looking for patterns, engaged in the real work of scientists and mathematicians” (Greg, October 19, 2015). A scientist who was a member of the PD faculty described that the student learning experience should involve “designing and developing within constraints [as this] models real world scenarios. .. realizing it is okay to learn from failure and that there isn’t just one right answer all the time” (Sophie interview, September 30, 2015).

The opportunity for students to develop and practice twenty-first century skills and dispositions was also included on over half of the concept maps. Participants listed specific skills, such as collaboration, communication, and perseverance. Expanding on this area in interviews, some connected these skills to career and life opportunities. As a TrMS math teacher described:

I think the end goal, what I would really want is students who can problem solve. … Life problems, work problems, I mean for years I’ve just thought employers just want employees who can think and take care of the problems at hand. Not have to be told, “Do this, do this, do this.” And so if you’re a problem solver you’re going to be a great employee. If you’re a problem solver you’re going to be a great inventor. (Olivia interview, January 26, 2015)

Less than one third of the participants included an explicit reference to STEM education as providing opportunities for all students to participate and be successful (Equ). Also, less than one third included ideas about technology (Tech), other than to write the word “technology” as part of STEM. We further discuss the low representation of these categories in the next section.

### Making sense of STEM education in different contexts

In this section, we address sub-question A regarding the themes educators in different professional contexts included in their conceptualizations of STEM education and the possible relationships between an individual’s conception of STEM education and the context in which she/he works. We first calculated the frequency of the inclusion of each theme for each context group. Table 4 shows that within context groups, different categories were more salient than others. We draw from our descriptions of the PD and school environments to consider potential relationships between the attributes of each context group’s STEM education work and the themes that were *most* or *least* commonly identified within that group.

#### PD faculty

Aside from the attributes common across all participants (interdisciplinary, instructional practices, and real-world problem solving), the statewide PD faculty, a group composed of people with a wide variety of backgrounds, commonly focused on broader concepts such as the global, societal value of STEM education (Val). This was also an overall theme of the summer STEM leadership institute developed by the PD faculty. The maps from the PD faculty also highlighted partnerships (Prtnr) between STEM professionals, teachers, and students. Claudia, a regional PD provider, indicated that STEM education benefits from community connections with “professionals in STEM, professionals related to STEM, informal science educators” and “benefits with support from parents, community professionals, and administrators” (Claudia concept map, May 7, 2015). The development of partnerships between schools and STEM professionals was addressed in multiple sessions during the institute, and two thirds of the PD faculty retained ideas about this attribute of STEM education when constructing their concept maps.

Ideas related to technology (Tech) were not commonly included on the PD faculty maps. Three of the four who included technology were people who worked most directly with it: the STEM school principal whose third- through eighth-grade students all had iPod touches or laptops, one of the business partners, and the district-level CTE director. On the fourth map that included technology, the strand of ideas was “STEM education ➔ multiple academic subjects ➔ technology [is] ill-defined” (Abel concept map, May 7, 2015). Abel’s notation is indicative of the confusion around what the T in STEM education means. At the institute, an invited presenter described how K-12 educators are uncertain about whether technology now means computer science, students’ and teachers’ use of information and communication technology (e.g., the internet; word processing and presentation tools), or tools more commonly found in CTE courses, such as 3D printers.

Forty-four percent of the PD faculty included an explicit relationship between STEM education and equitable learning opportunities (Equ), using phrases such as “teaching every child” (Marion concept map, May 7, 2015). Carlton expanded on this perspective: “It’s about the individual kid, not the industrial model of kids [coming through school]” (Carlton interview, September 30, 2015). Equity was a major theme of the institute, including a focused session at the beginning of the week and embedded in multiple sessions throughout.

Only one third of the PD faculty included standards (Stan), although standards received significant attention in a number of sessions during the institute. Also, less than half of this group included ideas about the student learning experience (StLE) or twenty-first century skills (21CS). The nature of the student experience in a STEM learning environment was modeled in a half-day session, although ideas about students’ opportunities to practice and develop twenty-first century skills were more implicit across sessions. The roles of PD faculty outside of the context of the institute might better explain why these three themes were not more frequently included on the concept maps of this group. We will discuss that in a subsequent section.

#### TrMS

As shown in Table 4, 50% or more of the participants in the TrMS group included attributes directly related to curriculum and instruction: interdisciplinary curriculum, ambitious instructional practices, attributes of students’ learning experiences, twenty-first century skills, standards, and real-world problem solving, in that order. These themes directly relate to elements of the professional development the teachers participated in for 2 years, where STEM design challenges were presented as a way to integrate standard-based mathematics and science content into existing curricula.

Over 50% of the participants from these two traditional middle schools also included ideas about various challenges associated with the implementation of STEM education (ChPrb). This reflects the constraints presented by their school contexts, including “time for planning” and “difficulties with creating in-depth integrated math and science problems.” Another challenge related to school structures that inhibited enacting the interdisciplinary, project-based curriculum units they were exploring in the TESI PD project. An eighth-grade science teacher explained:

The way our building is lined up or our schedule is we’re not in teams by any means. I mean my kids go off and see three different math teachers. So if it was ideal they’d have one math teacher, one science teacher, one humanities and we could do a little bit more of that integration, true integration. (Anthony interview, December 9, 2014).

Over 50% of the TrMS participants included references to standards (Stan) on their maps or mentioned these in interviews. Again, the context was important. Many of the comments reflected a negative relationship between the need to address standards and the desire to enact interdisciplinary, project-based curricula. Shawn, an eighth-grade teacher who had developed a new STEM elective course, commented on standards in this way:

I mean [this STEM course] is a great opportunity and I hope others get the chance and embrace it and run with it because I think it’s got a chance to be really successful and get some kids far better prepared for the real world than just learning back again state standards and stuff. I’ve probably been negative about state standards in my comments, and they’re important, but I don’t know that they focus enough on the STEM related skills, the integration of all this stuff to give kids successful opportunities to fulfill roles in business as problem solvers. (Interview, December 9, 2014)

These participants worked in two traditional middle schools in a district and state context where teachers were attempting to understand how to support students in meeting CCSS for mathematics and language arts, as measured by state achievement test data. Teachers were also just becoming familiar with the NGSS, both through the TESI project and other regional and district-level PD events. While the curricular units provided by the TESI project were aligned, the other instructional materials provided by the district were purchased prior to these new standards.

Ideas related to the access and opportunity for all students (Equ) were included on one third of the TrMS participants’ maps. The TESI summer institute was designed to help teachers recognize ways to support all students’ successful participation in STEM learning. For a week, students who had struggled with the content of their math or science courses joined their teachers in tackling engineering design challenges. Only four teachers explicitly identified this as an important feature of STEM education. A sixth-grade math teacher stated: “All kids bring skills, everyone’s good at something, no one’s good at everything” (Regan concept map, January 29, 2015) and a sixth-grade special education teacher constructed this strand on her map: “STEM education ➔ very inclusive ➔ kids of many levels can access something” (Brenda concept map, January 29, 2015). Others may have implied ideas about equity in other aspects of their concept maps, but there were no other explicit words or ideas either on maps or in interviews that we could code for this theme.

Three themes were seldom included or not included at all. Only one person from the TrMS group included ideas related to technology (Tech) and connecting it to “research skills.” This is not too surprising for traditional schools; one teacher pointed out the non-working Wi-Fi router on her classroom ceiling, and others commented that CTE classes were the only places where students could access technological tools. Students’ use of technology in the form of robotics was modeled in the summer PD but received little explicit attention other than that. Partnerships (Prtnr) and a broader value for STEM education (Val) did not appear on any concept maps in the TrMS group. While a variety of STEM professionals contributed to the activities of the summer institute, the development of partnerships in relation to supporting students’ interests in STEM careers and learning opportunities was not an explicit element of the PD.

#### RSA

Similar to the TrMS group, the participants from RSA most frequently included themes directly related to the classroom (IntDis, RWPS, InstPrac, StLE, 21CS; see Table 4). Also, over 50% of the RSA participants included partnerships (Prtnr) as an element of STEM education. This reflected a focus of their school philosophy, where building sustainable partnerships was supported with a half-time faculty position dedicated to cultivating business and academic partners to support student learning. The high school art teacher connected “relevance to real-world experiences” to “work-based learning and internships” (Josh concept map, October 15, 2015) and the principal represented this theme with a connection from STEM education to “extended learning opportunities and mentors” (Sandra concept map, June 4, 2015).

Similar to the other context groups, only 5 of the 13 participants from RSA included ideas about technology (Tech), although the technology was an explicit component of the school. A middle school history and language arts teacher who did include technology on his map explained why he positioned it as one of the major nodes: “I feel technology is embedded into everything. Because technology is just something that helps make the job easier” (Jason interview, November 4, 2014). The robotics and pre-engineering teacher discussed her vision for how technology should be integral to a STEM school:

I think for STEM education, space is very important and that’s one thing that we lack here. For maker space, fabrication projects, things like that. I mean both room as well as having the tools available. So C&C machines, we have a 3D printer but we haven’t been trained on using it yet. You know I mean just . . . any type of thing that you can think that a student might want to use to create. (Rachel interview, November 6, 2014)

The technology was of great importance to some of the RSA participants but not considered by the majority.

Ideas related to standards (Stan) were included on less than one third of the concept maps of the participants from RSA. While these teachers worked in the same state context as the TrMS teachers, they were located in a different district. More importantly, their school context differed. Teachers may have been more focused on the need to develop curriculum to address the school vision of interdisciplinary, project-based learning than to align with standards. However, the high school science teacher was very focused on the NGSS and developed two relevant strands on his concept map, one that connected STEM education ➔ integration ➔ 3D teaching ➔ science practices, concepts, and cross-cutting ideas, and another that connected STEM education to the K-12 Framework (National Research Council 2012). Alternately, the high school math teacher talked at length about how the pressures from testing specific standards at specific times was a roadblock to project-based learning: “I could develop a four-year program that would get kids to all standards, but the way it’s going now. .. we are trying to fill in skill gaps so how can we get into that real world stuff?” (Greg interview, October 19, 2015).

Finally, few of the RSA participants (15%) included or talked about opportunities for all students in STEM education (Equ). The principal wrote, “Do everything you can to support student success – make it happen” as the overarching concept on her map, and further explained in her interview:

You do everything you can to support student success and you make it happen. That’s what we’re after. Because every child can learn, every child wants to learn and be successful. And we just have practices and things in place in K-12 that separate out, that rank, and we know in our hearts and in our minds that not all students learn everything at the same pace, the same rate. It doesn’t mean they can’t learn or they won’t learn. (Sandra interview, June 4, 2015)

Also, the robotics teacher connected the curriculum ideas on her map to the challenge she faced in getting more girls interested in STEM areas (Rachel interview, November 6, 2014). Others did not specifically reference ideas related to equitable student opportunities. RSA opened as an inclusive STEM school and from conversations with RSA teachers separate from the data collection for this study, we know teachers are well aware of the need to support all kinds of students in STEM learning. However, based on the concept map data and interviews, teachers were not making explicit connections between the “most important ideas about STEM education” and opportunities for all students.

### Making sense of STEM education by role group

Given the multiple roles represented by the participants in this study, we next examined whether there would be notable similarities or differences in the conceptualizations of STEM education based on participants’ professional responsibilities (sub-question B; Table 5). Table 5 is organized in descending order of the most commonly included concept map themes by individual participants, making for an easy comparison between the global findings (reported in Table 3) and the frequency of inclusion by role group. Teachers of STEM-specific courses comprised the largest group, with 18 participants. Thus, it is not surprising that the most commonly included themes by individual and by context group are also those that STEM teachers most commonly included. Science, mathematics, technology, and CTE teachers are directly responsible for implementing the individually and/or collectively constructed vision of STEM education. They must identify or develop interdisciplinary curricula (IntDis) and determine how to bridge from in-school to real-world problems (RWPS). They understand that supporting students in the project- or problem-based learning experiences (StLE) will require instructional approaches that may differ from traditional, teacher-centered practices (InstPrac).

The interdisciplinary nature of STEM learning was by far the most salient feature for non-STEM teachers as well, and a significant focus by the administrators and external partners.

The art teacher from RSA explained:

I added art in there because I feel like that’s important. Turning it into STEAM. But like literally every single thing is intermingled. Like it’s a melting pot. All of it just goes together. Basically no matter what assignment, project, anything you pick you can connect every single one of these STEM or STEAM aspects into one another. (Brittany interview, November 10, 2014).

Real-world problem solving (RWPS) and ideas about instructional practices (InstPrac) were also included by the majority of non-STEM teachers, but the remaining themes were not consistently included. Many of the non-STEM teachers connected the need for an interdisciplinary approach to real-world problem solving yet faced challenges in connecting this approach to the standards they felt necessary to address. A sixth-grade special education teacher in the TrMS group explained that she wanted to bring in “Kind of authentic experiences and real-world [problems]” yet found that “it’s hard to integrate the 6th grade standards with STEM. I wish we had more time.” (Brenda interview, January 29, 2015).

School and district administrators all included ideas related to instructional practices, and most also included ideas about the student learning experience (StLE) and interdisciplinary curricula (IntDis). Administrators largely recognized most of the thematic elements of STEM education, except for the more global value (Val). In comparison, nearly all the external partners (regional PD providers and business or organization partners) included ideas related to this broader value of STEM education (Val) as well as connections to real-world problems (RWPS). Similar to all the role groups, the interdisciplinary nature of STEM curricula (IntDis) was included by most. External partners included external partnerships at a higher frequency than other groups.

As in the case of PD context, there is an indication in these data that the responsibilities of one’s specific job contribute to the elements of STEM education that are retained. Administrators, who tend to have responsibilities that relate to a large number of educational issues, gave explicit attention to numerous elements. Similarly, the broader outlook of the external partners, reflected in their attention to global values of STEM education in their concept maps, is consistent with their duties and responsibilities inside the STEM education system.

The ways in which the teacher participants made sense of STEM education was also consistent with their roles and responsibilities. Most teachers found interdisciplinary and real-world connections to be especially relevant. However, STEM teachers were also more likely to consider content standards, instructional approaches commonly associated with STEM education such as project-based learning, and twenty-first century skills in their conceptions. Non-STEM teachers were much more attentive to more general attributes of instruction, such as student-centered practices, engagement, and participation.

## Discussion

Those working with the implementation of STEM education are well aware that while core elements have been identified (Kelley and Knowles 2016; LaForce et al. 2014), there are still varying conceptions of what a STEM school or program entails. In this way, enacting STEM education entails innovation and motivates sensemaking. Our research shows that even when educators have similar professional learning experiences and/or work in the same contexts, they may make sense of what this innovation means quite differently. What is seen as most important to attend to or innovate around may differ in relation to professional roles and contexts.

Sensemaking provided a useful framework (Fig. 1) for considering the influence of institutional and professional contexts in shaping each educator’s construction of a plausible story of STEM education. Context appears to have some relationship with the ideas about STEM education noticed and retained by participants. This is most apparent in relation to partnerships, a key feature of the PD faculty work and of RSA. The identity of RSA as a STEM school supported teachers’ sensemaking about elements associated with STEM education such as interdisciplinary curricula, project-based learning, inclusion, and partnerships; these were part of the school vision statement. On the other hand, the professional identities of non-STEM teachers (e.g, English or history teachers) and STEM teachers shaped their individual meaning-making in relation to a STEM-focused curriculum. Teachers at the two middle schools were enacting STEM curricula in the context of a traditional middle school, with compartmentalized science and mathematics and a curricular focus aligned with statewide tests. Given these constraints, teachers in a more traditional school context may not take up ideas about STEM education that they encounter in professional learning experiences as readily as those in a STEM school context. The PD faculty worked in various professional contexts, with most in non-school settings. The STEM education ideas most salient to these scientists, business partners, and regional educators differed notably from those of STEM and non-STEM teachers.

In addition to the influence of institutional and organizational contexts, opportunities for collective reflection on the enactment of ideas associated with STEM education also contribute to an individual’s sensemaking (Davis 2003). Talking about actions involves “sensegiving,” which serves both to give information or feedback to others as well as an opportunity to “hear what one thinks” and further develop a plausible story (Weick et al. 2005, p. 416). For the TrMS teachers, there was an ongoing dialog with their colleagues, the PD providers, their instructional coaches, and their administrators. We can imagine that not only the traditional structures of the schools but also the differing ideas about and experiences with curriculum, instruction, and learning held by everyone involved in these conversations influenced the STEM education ideas the TrMS teachers selected and retained. Similarly, the PD faculty came together at least twice per year over 3 years to continually refine and co-construct their understandings about STEM education. Each drew upon relevant experiences from their professional roles and from educational research as they collectively developed a STEM education framework for each summer institute. At RSA, teachers met weekly to jointly develop curriculum and discuss student progress and school development. Teachers and administrators received feedback from the community of STEM professionals, parents, and district administrators, which also informed their conversations and subsequent sensemaking.

As shown in Table 3, our findings show the majority of educators in this study shared some common ideas about what is important for STEM education. However, identifying attributes and realizing these in practice are very different. For example, the interdisciplinary or integrated curriculum was the most identified theme across all concept maps. However, this may not be easily accomplished at many middle and high schools in the USA, as disciplinary skills and knowledge are often siloed, pacing guides determine time devoted to a given concept, and students move to different teachers in different groups. Opportunities to set up and engage in long-term STEM-related projects are constrained by these institutionalized practices as well as by space and equipment. Addressing this commonly identified attribute of STEM education will require tremendous creativity and resources.

Our analysis revealed other attributes that only a few included. The overall low representation of STEM education as an opportunity for *all* students is troubling. It may be that this was a concept educators considered but held distinct from STEM education. However, it has been apparent in education that when equity is not explicitly named and addressed, it is overlooked; Rodriguez (1997) termed this “the dangerous discourse of invisibility.” The inclusion of all students in STEM learning was emphasized in each of the contexts in this study yet failed to be retained as a salient attribute. The development of a STEM-literate citizenry and increased opportunities for all students to pursue STEM-related professions will require educators to explicitly address how students are included in or excluded from meaningful STEM learning.

## Conclusions

Our data suggest that professional roles and contexts influence the vision educators develop about STEM education. These results raise questions about the coherence of this innovation when people in the same school or district make sense of it in such different ways. Given the variety of institutionalized practices and contexts across schools, we are not convinced that a single worldwide definition of STEM education is critical. What we do see as essential is that those working in the same system, be it a department, school, or district, explore the common elements that are being attributed to STEM education and co-construct a vision that provides opportunities for all their students to attain STEM-related goals. Visioning, however, is insufficient, as what is envisioned and what is implemented are often very different. Educators must push on the status quo in areas of instruction, curriculum, learning opportunities, assessment, and school structures. Sensemaking as a collaborative, reflective, and iterative process can surface the differences and commonalities in people’s understandings to better ensure consistency in students’ learning opportunities across classrooms.

We propose that collective sensemaking through professional dialog be an explicit and ongoing activity when planning for and implementing STEM education. Supporting dialog among stakeholders from different contexts and professional roles is critical in order to ensure that diverse perspectives about the attributes for STEM teaching, learning, and curricula can be raised and discussed. For example, community members and policymakers may take a more global perspective focused on economic and societal implications. STEM and non-STEM teachers may focus on different aspects of the learning experience. Administrators are positioned to make sense of how individual teachers’ efforts contribute to student opportunities.

While it has been well established that professional development experiences, school vision statements, or readings about an innovation do not directly translate into the classroom and school practices (Penuel et al. 2008), explicitly identifying the ideas educators are and are not selecting and retaining can inform professional learning activities at local and larger scales. Further research is needed to understand more specifically what ideas educators notice, select, and retain about STEM education and how to support educators’ construction of plausible stories that promote a consistent vision of STEM education across a system.

## Footnotes

1 All names are pseudonyms
